# Detection of clonal plasma cells in POEMS syndrome using multiparameter flow cytometry

**DOI:** 10.1038/s41598-024-61034-1

**Published:** 2024-05-06

**Authors:** Arata Ishii, Shokichi Tsukamoto, Naoya Mimura, Yurie Miyamoto-Nagai, Yusuke Isshiki, Shinichiro Matsui, Sanshiro Nakao, Asuka Shibamiya, Yutaro Hino, Kensuke Kayamori, Nagisa Oshima-Hasegawa, Tomoya Muto, Yusuke Takeda, Tomoki Suichi, Sonoko Misawa, Chikako Ohwada, Koutaro Yokote, Satoshi Kuwabara, Chiaki Nakaseko, Hiroyuki Takamatsu, Emiko Sakaida

**Affiliations:** 1 Department of Hematology, Chiba University Hospital, Chiba, Japan; 2 Department of Endocrinology, Hematology, and Gerontology, Chiba University Graduate School of Medicine, Chiba, Japan; 3 Department of Transfusion Medicine and Cell Therapy, Chiba University Hospital, Chiba, Japan; 4 Department of Neurology, Chiba University Graduate School of Medicine, Chiba, Japan; 5 Department of Hematology, International University of Health and Welfare, Narita, Japan; 6 Department of Hematology, Institute of Medical, Pharmaceutical and Health Sciences, Faculty of Medicine, Kanazawa University, Kanazawa, Japan

**Keywords:** POEMS syndrome, Multiparameter flow cytometry, EuroFlow, Immunophenotype, Flow cytometry, Myeloma

## Abstract

POEMS syndrome (polyneuropathy, organomegaly, endocrinopathy, monoclonal protein [M-protein], and skin changes) is a rare systemic disorder characterized by various symptoms caused by underlying plasma cell (PC) dyscrasia. Detection of monoclonal PCs is mandatory for the diagnosis of POEMS syndrome; however, the usefulness of EuroFlow-based next-generation flow cytometry (EuroFlow-NGF) in POEMS syndrome for detecting monoclonal PCs in bone marrow (BM) and the gating strategy suitable for flow cytometry study of POEMS syndrome remain unknown. We employed EuroFlow-NGF-based single-tube eight-color multiparameter flow cytometry (MM-flow) and established a new gating strategy (POEMS-flow) to detect the monoclonal PCs in POEMS syndrome, gating CD38 broadly from dim to bright and CD45 narrowly from negative to dim compared to MM-flow. MM-flow detected monoclonal PCs in 9/25 (36.0%) cases, including 2/2 immunofixation electrophoresis (IFE)-negative cases (100%). However, POEMS-flow detected monoclonal PCs in 18/25 cases (72.0%), including 2/2 IFE-negative cases (100%). POEMS-flow detected monoclonal PCs with immunophenotypes of CD19^−^ in 17/18 (94.4%). In six cases where post-treatment samples were available, the size of the clones was significantly reduced after the treatment (*P* = 0.031). POEMS-flow can enhance the identification rate of monoclonal PCs in POEMS syndrome and become a valuable tool for the diagnosis of POEMS syndrome.

## Introduction

POEMS syndrome (polyneuropathy, organomegaly, endocrinopathy, monoclonal protein [M-protein], and skin changes) is a rare systemic disorder characterized by various symptoms that are a consequence of the presence of monoclonal plasma cells (PCs). In addition to the features indicated by the acronym, Castleman disease, sclerotic bone lesions, extravascular volume overload, papilledema, and thrombocytosis are known symptoms of POEMS syndrome^1^. One of the distinguishing features is a significant increase in serum vascular endothelial growth factor (VEGF) levels, which correlates with the disease activity^2,3^. POEMS syndrome is a chronic, progressive disorder with a poor prognosis. However, POEMS syndrome prognosis significantly improves with early diagnosis due to increased awareness of the disease, aggressive indications for autologous hematopoietic cell transplantation following high-dose melphalan, and the introduction of several novel agents similar to those used for treating multiple myeloma (MM), such as bortezomib-based or lenalidomide-based regimens^4,5^.

The diagnosis is made based on a composite of clinical symptoms and laboratory findings, and the international diagnostic criteria for POEMS syndrome are now extensively used^1,6,7^. Polyneuropathy and monoclonal plasma cell-proliferative disorder (PCD, almost always λ) are included as mandatory major criteria. In most cases of POEMS syndrome, immunofixation electrophoresis (IFE) of serum or urine detects M-protein although other laboratory findings, such as bone marrow (BM) histopathology, next-generation sequencing (NGS), and flow cytometry, are sometimes relevant in proving PCD^1^. A previous study reported that BM histopathology detected monoclonal PCs in two-thirds of cases^8^, but only less than 15% showed increased number of PCs^1^, indicating that BM examination is frequently nondiagnostic. We previously reported that NGS confirmed PC clones in BM in 36.7% of cases^9^. Furthermore, another study discovered that RNA-based immunoglobulin repertoire sequencing is a highly sensitive approach for proving the clonality of immunoglobulin light chain genes^10^. Previous studies reported that 6-color flow cytometry (FCM) confirmed PC clonality in less than 50% of POEMS syndrome cases^8,11^. EuroFlow-based next-generation flow cytometry (EuroFlow-NGF) is a highly sensitive tool widely used for assessing minimal residual disease (MRD) in MM^12,13^. However, its usefulness in detecting clonal PCs in POEMS syndrome has not been verified. Furthermore, the gating strategy suitable for POEMS syndrome has not been clarified. In this study, we aimed to verify the usefulness of EuroFlow-NGF-based multiparameter flow cytometry (MFC) in POEMS syndrome and explore suitable gating strategies to improve the diagnostic accuracy of POEMS syndrome.

## Methods

### Patients and BM sample preparation

This study enrolled patients with newly diagnosed or relapsed POEMS syndrome who were admitted to Chiba University Hospital from May 2019 to August 2021. POEMS syndrome diagnosis was made according to the international diagnostic criteria^7^. As part of routine clinical practice, BM samples of the patients were collected through BM examination. The collected BM samples were anticoagulated with ethylenediaminetetraacetic acid disodium salt dihydrate (EDTA-2Na), processed, and analyzed using MFC within 48 h. Normal control samples including 4 patients with newly diagnosed malignant lymphoma without BM involvement and 10 MM patients who achieved MRD-negative status after treatment were also analyzed.

This study was conducted under the Declaration of Helsinki principles and was approved by the ethics committee of Chiba University Graduate School of Medicine (Approval No. 1058). Before inclusion, each patient provided written informed consent.

### MFC analysis

The MFC analysis of BM samples was performed at SRL, Inc. (Tokyo, Japan), as described in a previous study^14^. In MM-flow panel analysis, which is a EuroFlow-NGF-based single-tube 8-color MFC method, BD FACSLyric Flow Cytometer (BD Bioscience, USA) and BD FACSuite software (BD Bioscience, USA) were used according to the manufacturers’ instructions. The sample preparation technique was based on a standardized lyse-wash-and-stain sample preparation protocol for EuroFlow-NGF (standard operating procedure [SOP]), and a single-tube 8-color antibody panel based on tube 2 of EuroFlow-NGF was used (CD138_V450_/CD27_V500_/CD38ME (multiepitope)_FITC_/CD56_PE_/CD45_PerCP-Cy5.5_/CD19_PE-Cy7_/cytoplasmic (Cy) Immunoglobulin (Ig) κ_APC_/CyIgλ_APC-H7_)^14^. According to previous reports^15,16^, PCs were identified by analyzing single cells after excluding debris and doublet cells with the use of forward scatter and side scatter.

Furthermore, Flow Cytometry Standard files provided by SRL, Inc were reanalyzed using FlowJo software (BD Bioscience, USA), and the PC gating strategy at Chiba University Hospital was modified as follows: (1) gating CD38 broadly with dim to bright, (2) gating CD45 narrowly with negative to dim, and (3) gating CD19-negative population selectively if there is an obvious population. Monoclonal PCs were defined if at least 20 cells were present and the κ/λ ratio was higher than four or less than 0.5 in any gating strategy.

### Statistical analyses

The patients’ characteristics were described using the median (range) for continuous variables and using the number of samples and frequency of each category for categorical variables. Spearman’s correlation coefficient was applied to evaluate the correction of paired data. Wilcoxon signed-rank test was used to compare the paired variables. All *P* values were obtained using a two-sided exact method, where *P* < 0.05 was considered statistically significant. All statistical analyses were performed using R statistical software version 4.1.2 (R Foundation for Statistical Computing).

## Results

### Patient characteristics

This study enrolled 25 patients (Table 1). The median age of the patients was 59 years (range 25–77); 48% of them were male, and 72% were newly diagnosed patients. IFE revealed the presence of M-protein of IgA-λ in 12 (48.0%), IgG-λ in nine (36.0%), IgA-κ in one (4.0%), IgG-κ in one (4.0%), and negative in two patients (8.0%), respectively. Two patients with negative M-protein by IFE were proven to have PCD using MFC (MM-flow). BM histopathology identified monoclonal PCs in only 25%. The median percentage of BM PCs was 1.7% (range 0.6–15.3%), with only one patient (4%) exceeding 10%. Serum VEGF levels (normal range ≤ 38.3 pg/mL) increased significantly in all patients, with a median of 3440 pg/mL (range 747–9060). All the patients had at least one laboratory finding to prove PCD among IFE, BM histopathology, or flow cytometry. BM histopathology and papilledema were not examined in one patient each.

**Table 1:** Patient characteristics.

Characteristic	N = 25^a^
Age	59 (25–77)
Male sex	12 (48%)
Immunofixation electrophoresis	
IgA-κ/IgA-λ	1 (4.0%)/12 (48%)
IgG-κ/IgG-λ	1 (4.0%)/9 (36%)
Negative	2 (8.0%)
Bone marrow plasma cells (%)	1.70 (0.60–15.30)
Positive bone marrow biopsy (%)	6 (25%)^b^
VEGF (serum) (pg/mL)*	3440 (747–9060)
POEMS features	
Polyneuropathy	25 (100%)
Organomegaly	20 (80%)
Endocrinopathy	17 (68%)
Monoclonal gammopathy	25 (100%)
Skin changes	22 (88%)
Castleman	4 (16%)
Sclerotic bone lesions	16 (64%)
Extravascular volume overload	23 (92%)
Papilledema	10 (42%)^b^
Thrombocytosis/polycythemia	19 (76%)
Newly diagnosed/relapsed	18 (72%)/7 (28%)

VEGF, vascular endothelial growth factor

*Normal range ≤ 38.3 pg/mL.

^a^Median (Range); n (%).

^b^N = 24.

### POEMS-flow gating strategy setup

**Figure 1:**
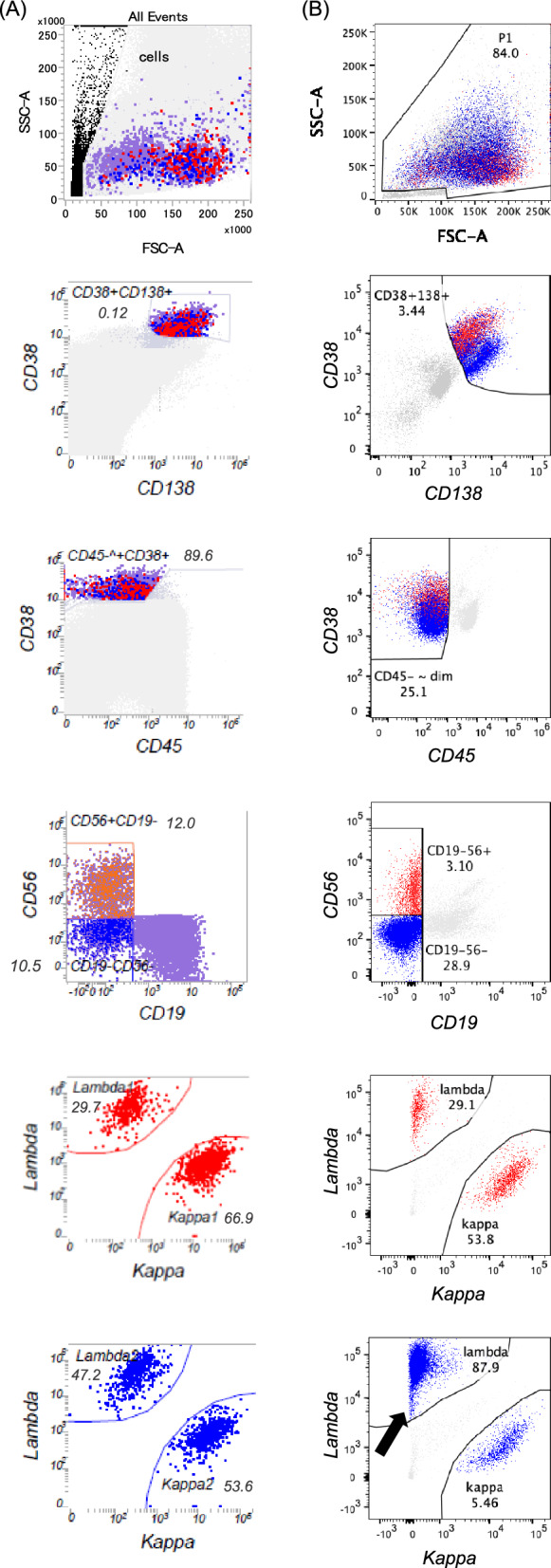
Different gating strategies in MM-flow and POEMS-flow. Gating strategies in (**A**) MM-flow and (**B**) POEMS-flow in representative cases of POEMS syndrome are shown. In MM-flow, CD38^+^ (brightly positive)/CD138^+^/CD45^−~+^ (negative to positive)/CD19^−^/CD56^±^ PCs are analyzed in a panel of cytoplasmic immunoglobulin (cy-Ig) light chain expression and the light chain-restricted PCs are determined to be monoclonal PCs. In POEMS-flow, CD38 is gated broadly with dim to bright and CD45 is gated narrowly with negative to dim. CD38^+^ (broadly positive)/CD138^+^/CD45^−^(narrowly negative)/CD19^−^/CD56^±^ PCs are further analyzed in the panel of cy-Ig light chain expression and the light chain-restricted PCs are determined to be monoclonal PCs. In the representative case of POEMS syndrome shown in this figure, monoclonal PCs are not identified by (**A**) MM-flow; however, (**B**) POEMS-flow detects monoclonal PCs (CD38^+^/CD138^+^/CD45^−^/CD19^−^/CD56^−^/λ^+^ population indicated by an arrow). (**A**) The number indicates the percentage of CD38^+^/CD138^+^ cells in single cells, CD45^−~+^/CD38^+^/CD138^+^ cells in CD38^+^/CD138^+^ cells, CD19^−^/CD56^±^ cells in CD45^−~+^/CD38^+^/CD138^+^ cells, and the light chain-restricted PCs in CD38^+^/CD138^+^/CD45^−~+^/CD19^−^/CD56^±^ PCs, respectively. (**B**) The number indicates the percentage of CD38^+^/CD138^+^ cells in single cells, CD45^−^/CD38^+^/CD138^+^ cells in CD38^+^/CD138^+^ cells, CD19^−^/CD56^±^ cells in CD45^−^/CD38^+^/CD138^+^ cells, and the light chain-restricted PCs in CD38^+^/CD138^+^/CD45^−~+^/CD19^−^/CD56^±^ PCs, respectively. The numbers in the figures represent the percentages per single cell for each cell population. The size of monoclonal PCs identified by (**B**) POEMS-flow is 0.22% per single cell, and the κ/λ ratio is 0.062.

In a previous study, 6-color FCM revealed the presence of both abnormal, λ light chain-restricted PCs and normal, polytypic PCs, as indicated by differential CD45 expression in patients with POEMS syndrome^8^. Furthermore, our single-cell RNA sequencing study revealed that CD38 and CD19 were downregulated in monoclonal POEMS PCs^17^. Based on these findings, we developed a new gating strategy (POEMS-flow) by modifying the gating of CD38 and CD45 from MM-flow (Fig. 1). In MM-flow, we analyzed CD38^+^ (brightly positive)/CD138^+^/CD45^−~+^ (negative to positive)/CD19^−^/CD56^±^ PCs in the panel of cytoplasmic immunoglobulin (cy-Ig) light chain expression and light chain-restricted PCs were identified as monoclonal PCs (Fig. 1A). However, in POEMS-flow, CD38 was gated broadly with dim to bright and CD45 was gated narrowly with negative to dim. CD38^+^ (broadly positive)/CD138^+^/CD45^−^ (narrowly negative)/CD19^−^/CD56^±^ PCs were further analyzed in a panel of cy-Ig light chain expression, and light chain-restricted PCs were identified as monoclonal PCs (Fig. 1B). This optimized gating strategy successfully identified monoclonal PCs within a background of polyclonal PCs.

### Identification rate of monoclonal PCs in POEMS syndrome by MM-flow and POEMS-flow

Next, we analyzed all samples using both MM-flow and POEMS-flow and compared them in the identification rate of monoclonal PCs in POEMS syndrome. Monoclonal PCs were detected in 9 (36.0%) and 18 (72.0%) of the 25 cases using MM-flow and POEMS-flow, respectively. Among the 23 cases with positive IFE, monoclonal PCs were detected in 7 (30.4%) and 16 (69.6%) cases using MM-flow and POEMS-flow, respectively. Two IFE-negative cases detected monoclonal PCs in both MM-flow and POEMS-flow. Among the 17 newly diagnosed cases, monoclonal PCs were detected in 7 (38.9%) and 13 (72.2%) cases using MM-flow and POEMS-flow, respectively. Among the 8 relapsed cases, monoclonal PCs were detected in 3 (42.9%) and 5 (71.4%) cases using MM-flow and POEMS-flow, respectively. All normal control samples had no monoclonal PCs detected in MM-flow or POEMS-flow. In MM-flow, seven (77.8%) of the monoclonal PCs were of the λ-type and two (22.2%) were of the κ-type, whereas in POEMS-flow, 16 (88.9%) were of the λ-type and two (11.1%) were of the κ-type. These were consistent with the light chains detected using IFE, except in two IFE-negative cases. The size of the detected clones in both the methods was not statistically different, and the median size of the clones was small, 0.03% (range 0.008–3.782%) for MM-flow and 0.032% (range 0.006–4.720%) for POEMS-flow (Table 2). The clone size was not correlated with serum VEGF levels in either MM-flow (r = 0.08) or POEMS-flow (r = 0.11).

**Table 2:** The size of the detected clones in MM-flow and POEMS-flow.

	N	Monoclonal plasma cells/single cells (%)^a^
MM-flow	9	0.030 (0.008–3.782)
POEMS-flow	18	0.032 (0.006–4.720)

^a^Median (range); n (%).

### The immunophenotype of the detected monoclonal PCs using POEMS-flow

**Figure 2:**
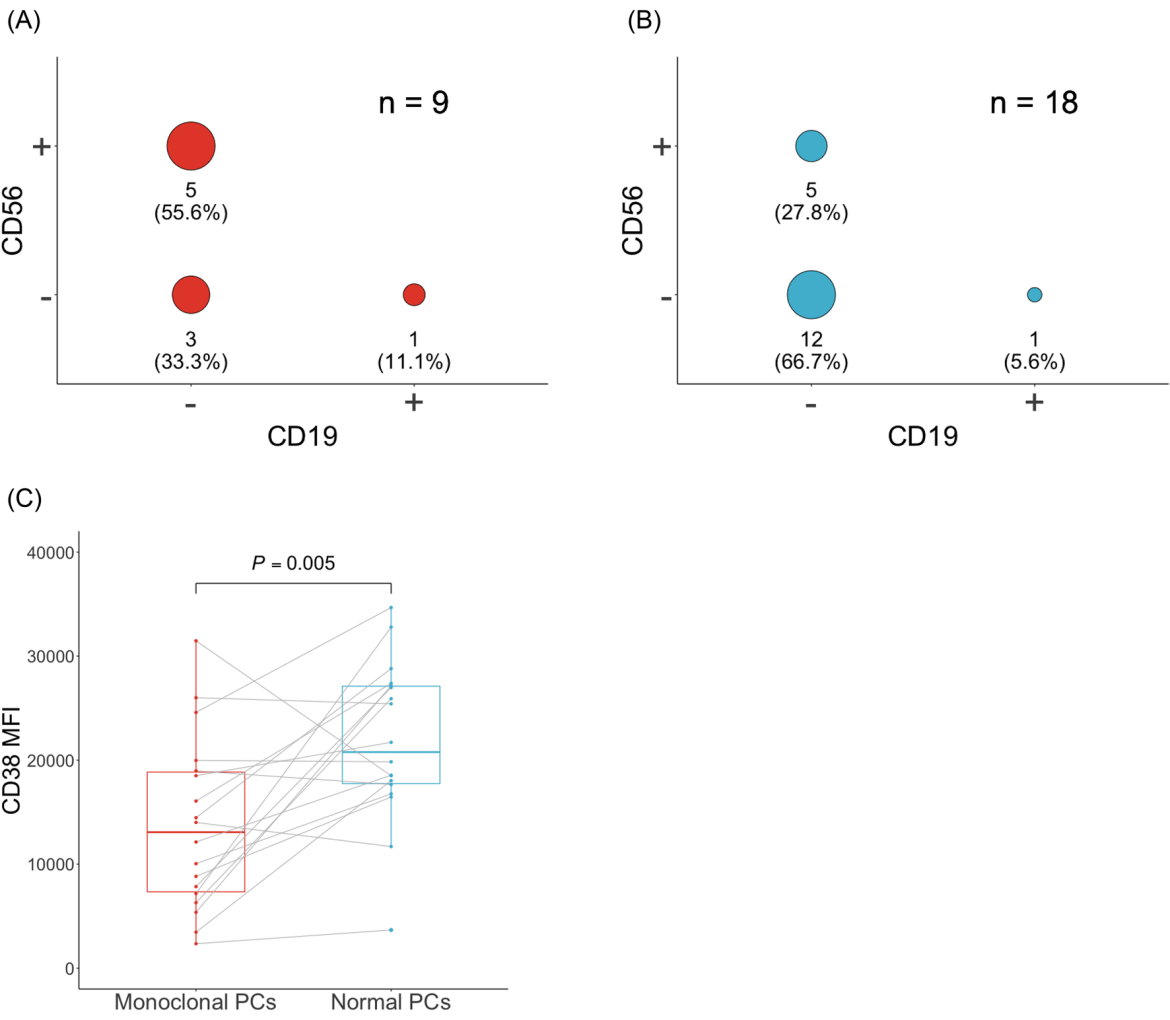
The immunophenotype of the monoclonal plasma cells in POEMS syndrome. The immunophenotype of CD19 and CD56 in the identified clonal PCs using (**A**) MM-flow (n = 9) and (**B**) POEMS-flow (n = 18) are shown. The size of the circle in the figure indicates the number of cases with different immunophenotypes of CD19 and CD56 (CD19^−^/CD56^+^ or CD19^−^/CD56^−^ or CD19^+^/CD56^−^). (**A**) Five (55.6%) were CD19^−^/CD56^+^ and three (33.3%) were CD19^−^/CD56^−^ in MM-flow. (**B**) Five (27.8%) were CD19^−^/CD56^+^ and 12 (66.7%) were CD19^−^/CD56^−^ in POEMS-flow. (**C**) A comparison of CD38 mean fluorescence intensity (MFI) between monoclonal and normal plasma cells (PCs) analyzed using POEMS-flow is shown. The CD38 MFI of monoclonal PCs was significantly lower than that of normal PCs (*P* = 0.005), with median MFI values of 13,074.5 (range 2355–31,472) for monoclonal PCs and 20,778 (range 3674–34,672) for normal PCs.

In both MM-flow and POEMS-flow, the immunophenotype of the detected monoclonal PCs was CD19 negative in all but one case (CD19^+^/CD56^−^). Five (55.6%) were CD19^−^/CD56^+^ and three (33.3%) were CD19^−^/CD56^−^ in MM-flow (Fig. 2A), whereas five (27.8%) were CD19^−^/CD56^+^ and 12 (66.7%) were CD19^−^/CD56^−^ in POEMS-flow (Fig. 2B), indicating that the immunophenotype of monoclonal PCs in POEMS syndrome was similar to that in MM. To examine the difference in CD38 expression between monoclonal and normal PCs in POEMS syndrome, we analyzed CD38 mean fluorescence intensity (MFI) in 18 cases where monoclonal PCs were detected by POEMS-flow. Normal, polyclonal PCs were defined as CD38^+^/CD138^+^/CD19^+^/CD56^−^/κ or λ (not restricted) population in each sample. The CD38 MFI of monoclonal PCs was significantly lower than that of normal PCs (*P* = 0.005, Fig. 2C), with median MFI values of 13,074.5 (range 2355–31,472) for monoclonal PCs and 20,778 (range 3674–34,672) for normal PCs. In total, 13 (72.2%) of 18 cases where POEMS-flow discovered monoclonal PCs exhibited lower CD38 MFI of monoclonal PCs, with 10 cases (76.9%) being newly diagnosed patients. The CD27 MFI of monoclonal PCs was significantly lower than that of normal PCs (*P* = 0.007), with median MFI values of 2500 (range 235–8524) for monoclonal PCs and 3111.5 (range 1867–5387) for normal PCs. However, gating CD27 negative to dim in addition to POEMS-flow did not improve the detection rate of monoclonal PCs.

### Change in the size of the clones identified by POEMS-flow after treatment

**Figure 3:**
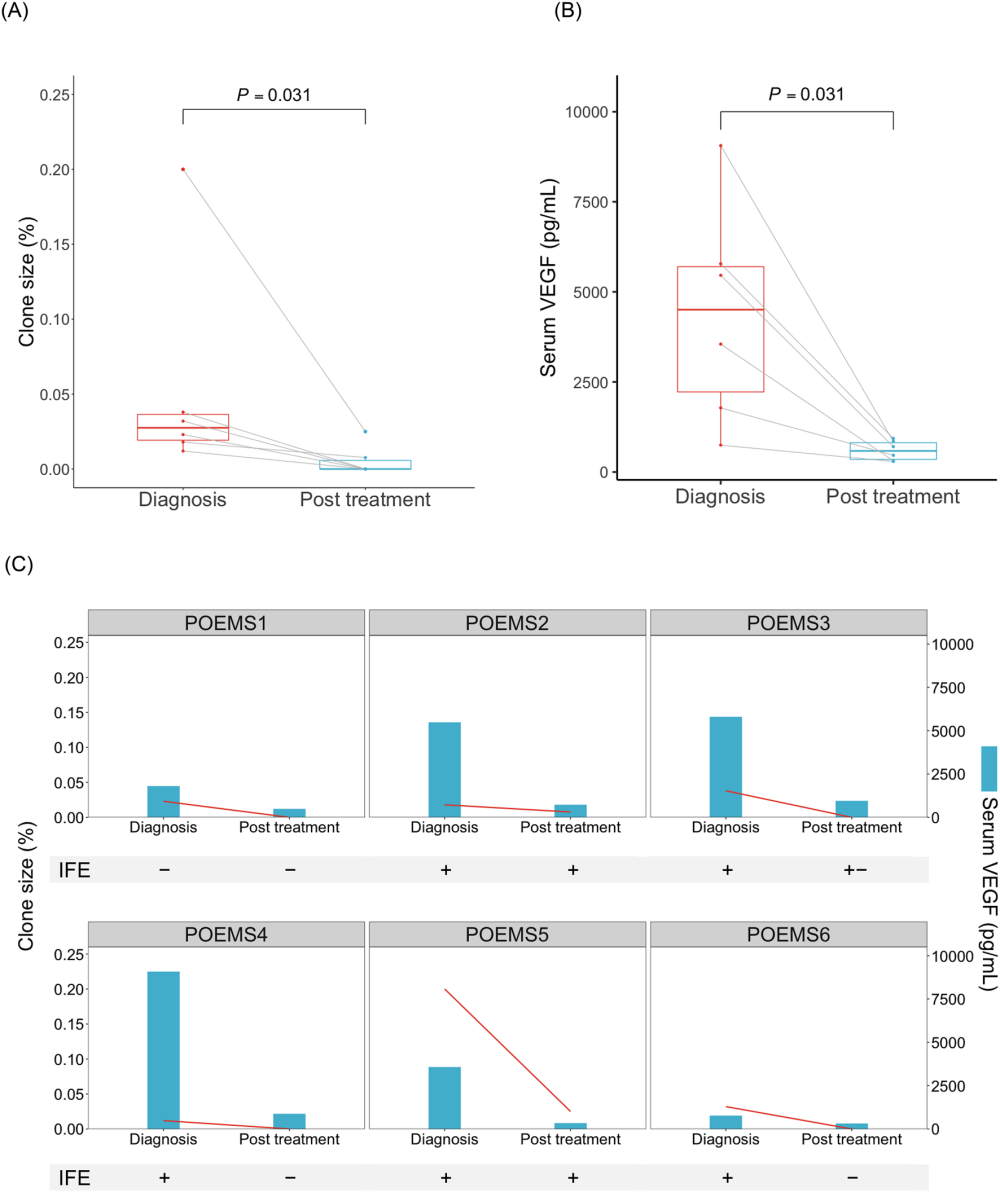
Change in the size of clones detected by POEMS-flow after treatment. Six cases in which monoclonal PCs were identified at the time of diagnosis by POEMS-flow and the available post-treatment samples are evaluated. (**A**) Change in the size of clones detected using POEMS-flow after treatment. The size of the clones was significantly reduced after the treatment (*P* = 0.031) in all six patients. (**B**) Change in serum vascular endothelial growth factor (VEGF) levels after treatment. Serum VEGF levels were significantly decreased after treatment (*P* = 0.031) in all six patients. (**C**) Changes in the size of clones detected using POEMS-flow, serum VEGF levels, and the status of M-protein using immunofixation electrophoresis (IFE) for each patient after treatment.

To investigate whether POEMS-flow was valuable for the follow-up of MRD during the clinical course, we assessed the change in the size of the clones in six cases in which monoclonal PCs were detected at the time of diagnosis by POEMS-flow and for which post-treatment samples were available. The median time from diagnosis to post-treatment sample collection was 155 days (range 73–245). Five patients received bortezomib and dexamethasone therapy, with a median number of five cycles (range 3–8). One patient received four cycles of lenalidomide and dexamethasone therapy. The size of the clones was significantly reduced after the treatment (*P* = 0.031) in all six patients (Fig. 3A). The median clone size during diagnosis was 0.0275% (range 0.012–0.2%), and no clones were detected after treatment, except in two cases (0.025% and 0.0077%). Serum VEGF levels were also significantly decreased after treatment in all six patients (*P* = 0.031; median, 4505 pg/mL at diagnosis vs. median, 585 pg/mL after treatment) (Fig. 3B). M-protein by IFE was negative in one patient both at the time of diagnosis and after treatment, became negative after treatment in three patients, and remained positive in two patients. In three patients whose monoclonal PCs had become undetectable using POEMS-flow after treatment (POEMS3, 4, and 6), M-protein using IFE also became negative or indeterminate (Fig. 3C). Overall, the clone size detected by POEMS-flow, serum VEGF levels, and the status of M-protein behaved similarly before and after treatment.

## Discussion

This study evaluated the usefulness of EuroFlow-NGF-based MFC in detecting clonal PCs in POEMS syndrome in an era when highly sensitive EuroFlow-NGF is widely used for MRD assessment in MM. We further proposed optimized gating strategies to enhance the identification rate of monoclonal PCs in POEMS syndrome. MM-flow was established based on EuroFlow-NGF for MRD assessment in MM^14^ and was not designed for detecting clonal PCs in POEMS syndrome. Therefore, we first set up a new gating strategy (POEMS-flow) by modifying the gating of CD38 and CD45 from MM-flow (Fig. 1) based on the important findings from our and other previous studies: (1) polyclonal and monoclonal PCs had differential CD45 expressions in 6-color FCM analysis^8^, (2) CD38 and CD19 mRNA expression was significantly downregulated by single-cell analysis^17^. Several reports have verified the usefulness of conventional flow cytometry analysis of BM samples in detecting clonal PCs in POEMS syndrome; however, the detection rate was insufficient as two studies reported that 6-color MFC detected monoclonal PCs in 11/62 (17.7%)^11^ and 7/17 (41.2%)^8^ of newly diagnosed patients, respectively. Interestingly, despite taking advantage of EuroFlow-NGF-based MFC, monoclonal PCs were detected only in 9/25 (36.0%) cases in our study, which was not much different from conventional FCM. In contrast, our optimized POEMS-flow significantly improved the detection rate of monoclonal PCs (18/25 cases, 72.0%), indicating that POEMS-flow is more suitable for detecting clonal PCs in POEMS syndrome than MM-flow.

Despite the establishment of international criteria^7^, POEMS syndrome diagnoses can be difficult due to the mixture of various clinical and laboratory features. To prove monoclonal PCD, which is a mandatory major criteria for POEMS syndrome diagnosis^7^, IFE test is a standard method and a previous study reported 87/99 (87.9%) of patients with POEMS syndrome had positive IFE of either serum or urine^1^, indicating that there are some IFE-negative cases in POEMS syndrome. Some patients with POEMS syndrome were clinically diagnosed without any evidence of PCD, and their symptoms improved with treatment^18,19^. Recent epidemiological surveys reported that PCD was not proven in 11% of patients with POEMS syndrome^20^. Furthermore, a descriptive study of 12 POEMS syndrome patients without proven PCD reported that there were not many differences in clinical manifestations from POEMS syndromes with proven PCD, and all cases had improved clinical symptoms and decreased serum VEGF levels after treatment^21^. In our study, two IFE-negative cases were included, and monoclonal PCs in BM were detected using MM-flow and POEMS-flow in both cases. POEMS syndrome without proven PCD has not been widely accepted yet and PCD detection is crucial for POEMS syndrome diagnosis, providing appropriate and effective treatment for patients with suspected POEMS syndrome. Careful analysis of immunohistochemical staining of biopsy specimens from osteosclerotic lesions and BM was reported to be useful in POEMS syndrome diagnosis^1,8^. It is especially reported that immunohistochemical staining of bone marrow architecture, in particular on “plasma cell rimming,” is more sensitive than 6-color flow^8^. POEMS-flow may be effective in proving PCD, even if it is IFE-negative, and when combined with various examinations, it may improve the diagnostic accuracy of POEMS syndrome.

In this study, the immunophenotype of the detected monoclonal PCs in POEMS syndrome using POEMS-flow was CD19^−^/CD56^+^ in 5/18 (27.8%), CD19^−^/CD56^−^ in 12/18 (66.7%), and CD19^+^/CD56^−^ in 1/18 (5.6%) cases, respectively (Fig. 2B). Normal PCs usually express CD19^+^/CD56^−^, but MM PCs are typically negative for CD19 and positive for CD56 in 60–75% of cases^22^. CD19 expression in PCs of monoclonal gammopathy of undetermined significance is lower than in normal PCs but higher than in MM PCs, and loss of CD19 has been associated with tumor progression^23^. Several reports referring to the immunophenotype of POEMS syndrome also reported that CD19 was negative^8,11,24,25^, which is consistent with this study, indicating that the monoclonal PCs in POEMS syndrome are immunophenotypically distinct from normal polytypic PCs. On the other hand, one case with CD19^+^ monoclonal PCs was detected in this study (Fig. 2A,B). Although CD19 negativity is one of the most frequent aberrancies in clonal PCs, CD19^+^ clonal PCs have also been reported. Therefore, gating strategies to detect monoclonal PCs should not be restricted to the CD19^−^ compartment, but the CD19^+^ compartment always needs to be checked for potential clonality. Also, this study revealed that CD38 MFI is significantly lower in monoclonal PCs of POEMS syndrome compared to normal PCs (Fig. 2C). Previous studies have discovered that CD38 expression is also reduced in MM PCs although it is highly expressed in normal PCs^22,23^. MM-flow, an MM-focused gating strategy detected monoclonal PCs in 9/25 (36.0%) POEMS syndrome cases. In contrast, POEMS-flow, gating CD38 more broadly from dim to bright, was detected in twice the number of cases (18/25; 72.0%). Further, our single-cell RNA sequencing study revealed that CD38 expression was significantly downregulated in the monoclonal POEMS PCs compared to normal PCs^17^. Therefore, despite the reduced CD38 expression in POEMS PCs, POEMS-flow is suitable for detecting clonal PCs in POEMS syndrome. Furthermore, this method may also be effective for detecting clonal PC in some cases of MM, although further studies are needed to evaluate this point.

This study has several limitations. First, this was a single-center study, and further investigations of more samples from a multi-center are needed to evaluate and validate the usefulness of the optimized POEMS-flow proposed in this study. Second, MM-flow is a EuroFlow-NGF-based single-tube 8-color MFC that uses a different technique from two-tube EuroFlow-NGF. However, MM-flow has been validated to fulfill the criteria for the NGF method, which includes routine assessment of more than 5 million mononuclear cells to estimate MRD and a sensitivity of one in 10^5^ cells or higher^14,26^. Then we applied MM-flow and further evaluated the usefulness of MM-flow in detecting clonal PCs in POEMS syndrome in this study. Finally, our POEMS-flow detected monoclonal PCs in 72% of the patients with POEMS syndrome; however, even with this optimized gating strategy, monoclonal PCs were not detected in approximately 30% of cases, which was lower than the rate of detecting M-protein by IFE. This could be because cases with no detectable bone marrow plasma cells may have solitary or multiple solitary plasmacytomas, which is one of the limitations to detect clonal PCs using bone marrow samples.

Conclusively, this study evaluated the sensitivity of EuroFlow-NGF-based MFC in detecting clonal PCs in POEMS syndrome and demonstrated the usefulness of the optimized POEMS-flow, gating CD38 broadly from dim to bright and CD45 narrowly from negative to dim compared to EuroFlow-NGF-based MFC. This strategy needs to be validated in a larger cohort. This preliminary data indicates that POEMS-flow could improve the identification rate of monoclonal PCs in POEMS syndrome and become a valuable tool for diagnosing POEMS syndrome.

## Data Availability

The datasets generated during and/or analyzed during the current study are available from the corresponding author upon reasonable request.
